# Active screening and patient-placement and cohort-placement strategies to decrease carbapenem-resistant gram-negative bacilli colonization and infection in pediatric patients: A 5-year retrospective observational study in China

**DOI:** 10.1017/ice.2023.20

**Published:** 2023-10

**Authors:** LJ Yin, LY He, GP Lu, Y Cao, LS Wang, XW Zhai, CQ Wang

**Affiliations:** 1 Department of Nosocomial Infection Control, Children’s Hospital of Fudan University, Shanghai, China; 2 Clinical Microbiology Laboratory, Children’s Hospital of Fudan University, Shanghai, China; 3 Pediatric Intensive Care Unit, Children’s Hospital of Fudan University, Shanghai, China; 4 Neonatal Intensive Care Unit, Children’s Hospital of Fudan University, Shanghai, China; 5 Department of Neonatology, Children’s Hospital of Fudan University, Shanghai, China; 6 Department of Hematology, Children’s Hospital of Fudan University, Shanghai, China

## Abstract

Carbapenem-resistant gram-negative bacilli (CR-GNB) colonization screening was initiated across high-risk departments (PICU, NICU, neonatal wards, and hematology departments) in January 2017, and several CR-GNB cohort and patient-placement strategies were introduced throughout the hospital in January 2018. The colonization and infection rates decreased to varying degrees from 2017 to 2021.

Carbapenem-resistant gram-negative bacilli (CR-GNB) are a serious cause of healthcare-associated infections.^1^ To contain antimicrobial resistance in China, the “two steps and two hands” strategy was implemented. Promoting the rational use of antimicrobial agents and precise control are the 2 “steps.” Optimizing infrastructure and cultivating professional teams are the 2 “hands.”^2^ However, controversy continues regarding the most pragmatic and evidence-based approach to prevent CR-GNB cross transmission.^3^ The large ward-room setting and relatively insufficient medical resources in China made CR-GNB single-room isolation and cohort administration impractical. To study the impact of active screening and patient cohort-placement strategies on CR-GNB colonization and infection, we conducted this 5-year retrospective study.

## Methods

### Study design and intervention

The Children’s Hospital of Fudan University is an 800-bed, tertiary-care, teaching hospital comprising 3 intensive care units (ICUs, each with 40–60 beds), 12 surgical and 16 medicine wards. Patients under 18 years of age are considered pediatric. Most patients are cared for in 5-bed rooms in medical and surgical wards. ICUs are organized in open spaces, with a nurse-to-patient ratio of 2.5.

A project to decrease CR-GNB nosocomial infection incidence was initiated in 2017. CR-GNB colonization screening in the pediatric ICU, neonatal ICU, neonatology and hematology departments (hereafter referred to as high-risk departments) was implemented in January 2017.^4^ All CR-GNB–positive patient isolation and cohort-placement strategies were executed across the entire hospital beginning in January 2018. Other basic infection precaution bundle measures were implemented before 2017.

The study was approved by the Ethics Committee of the Children’s Hospital of Fudan University, Shanghai, China (no. 2021-372).

### Basic infection precaution bundle measures

Contact precautions, including hand hygiene, surgical masks, hats, use of gowns and gloves along with patient and staff cohort placement, were implemented once CR-GNB was detected in a patient. Hand hygiene adherence and environmental cleaning and disinfection qualification rates (ie, number of environmental samples without positive culture divided by the total number of environmental samples) were monitored. Previously published definitions of hospital-associated infection and colonization were used.^5,6^

### Active surveillance program

An intestinal and upper-respiratory tract CR-GNB active screening program was implemented in 2017. This program included pharyngeal and rectal swab (stool screening for hematology patients) within 48 hours after patient admission and once per week thereafter during admission. Repetitive screening of known CR-GNB colonized patients was conducted weekly during hospitalization.

### Patient isolation and cohort placement program

Beginning in January 2018, 4 types of patient placement were used for patients known to be colonized or infected with CR-GNB. Single-room placement (type A) refers to 1 patient in 1 room. Same-room placement (type B) refers to placement of patients who were infected or colonized with the same CR-GNB in the same room. Same-area placement (type C) means patients with the same CR-GNB were placed in cohorts in the same area of a multibed room with use of partition barriers. No cohort placement (type D) refers to placing patients with or without CR-GNB or with different CR-GNB in the same room without any attempt to group patients with the same CR-GNB status. Curtains were used between beds.

### Microbiological methods

CR-GNB culture, identification, and antimicrobial susceptibility tests were performed as previously described.^4^ Clinical information for patients with CR-GNB was systematically retrieved from electronic medical records.

### Statistical analysis

All data are expressed as rates for categorical variables. Comparison of rates between groups were performed using the χ^2^ test; *P* <0.05 was regarded as statistically significant. All statistical analyses were performed using SPSS version 21.0 software (IBM, Armonk, NY).

## Results

### CR-GNB–positive patients and use of isolation and cohorting strategies

**Fig. 1.**
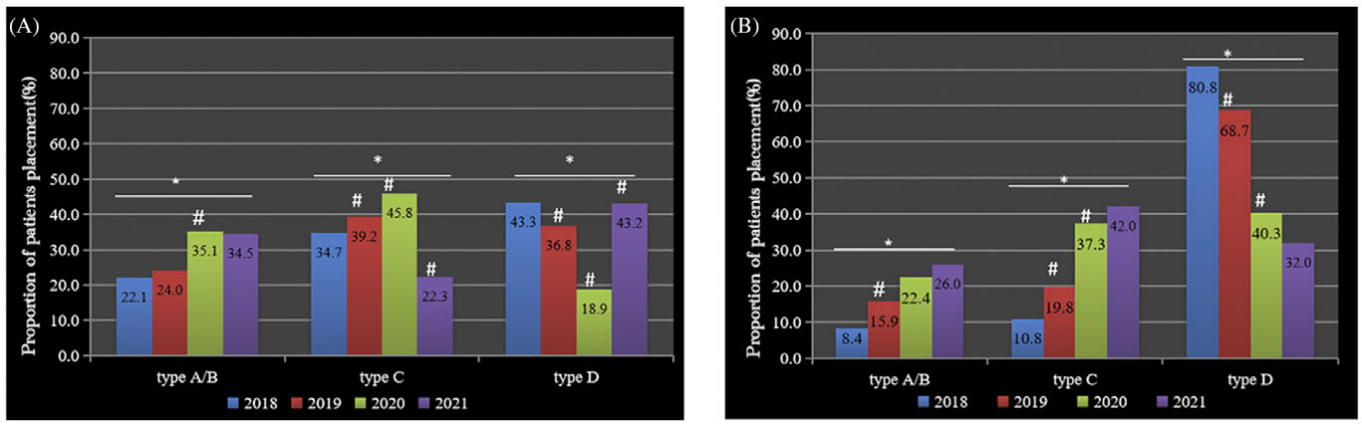
Percentage of patients with carbapenem-resistant gram-negative bacilli (CR-GNB) cared for using each of 4 isolation and cohort-placement strategies: 2018–2021(%) Various CR-GNB patient placements were performed in (A) high-risk departments (pediatric ICU, neonatal ICU, neonatal and hematology departments) and (B) non–high-risk departments. The proportion of patients managed using placement type A (isolation) or B (cohort placement) increased in high-risk departments and non–high-risk departments. Note. Type A, single-room placement; type B, same-room placement; type C, same-area placement; type D, no cohort placement. *P <.05, comparison of patient placement type between years. ^#^ *P* <0.05 compared with the previous year.

Between 2017 and 2021, 5,078 CR-GNB–positive patients were identified: 4,161 in high-risk departments and 917 in non–high-risk departments. Among them, 2,016 were identified by clinical culture, 2,711 by screening, and 351 by both screening and positive clinical culture. Also, 3,948 CR-GNB–positive patients (1,208 by clinical culture, 2,438 by screening, and 302 by both screening and positive clinical positive) received different types of patient placement between 2018 and 2021. The use of placement types A and B increased in high-risk and non–high-risk departments. Compared with the previous year, the use of placement types A and B increased in 2020 and 2019 in high and non–high-risk departments, respectively (all *P* < .05) (Fig. 1 and Supplementary Table S1 online).

We detected no significant change in the hand hygiene compliance rate or the environmental cleaning and disinfection qualification rate (*P* >.05) (Supplementary Table S2 online).

### CR-GNB colonization prevalence in high-risk departments

**Fig. 2.**
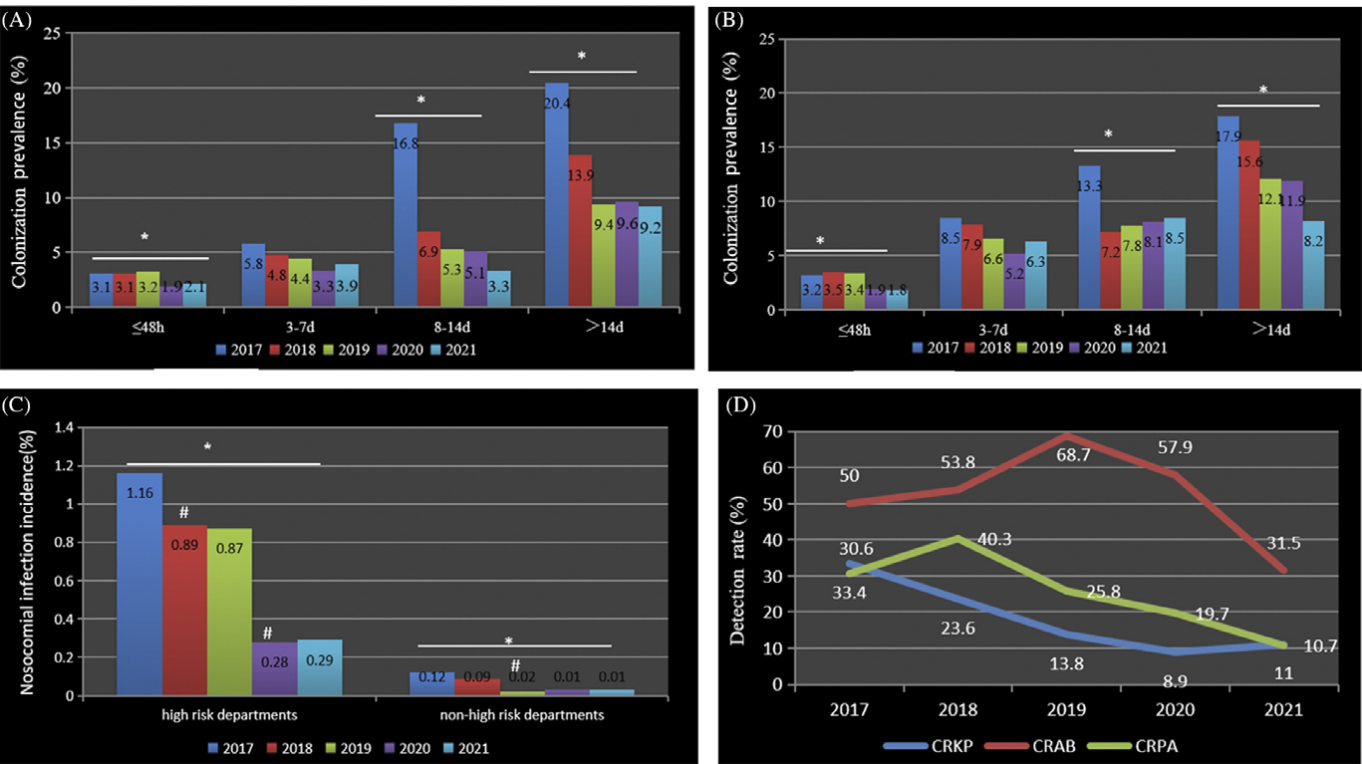
Carbapenem-resistant gram-negative bacilli (CR-GNB) colonization prevalence, nosocomial infection incidence and detection rate (%). The (A) upper respiratory and (B) intestinal tract CR-GNB colonization prevalences, (C) nosocomial infection incidence, and (D) detection rate decreased (*P* <0.001). *P < 0.05, comparison between years. ^#^ *P* < 0.05, compared with the previous year. The nosocomial incidence rate is the number of new nosocomial infection cases divided by the number of patients in the hospital during the observation period×100%. The CR-GNB detection rate is the number of CR-GNB positive clinical isolates divided by the number of gram-negative bacilli–positive clinical isolates×100%.

From 2017 to 2021, >20,000 patients were screened >50,000 times in total, with detection of 2,711 CR-GNB–positive patients. Active screening was mainly concentrated within 48 hours of admission (Supplementary Table S3 online). Each year, CR-GNB colonization prevalence in the upper-respiratory tract and the intestinal tract significantly increased, with an increased length of hospital stay. However, colonization prevalence decreased from 2017 to 2021 among patients in all duration-of-hospitalization categories except the 3–7-day hospitalization group (*P* < .001) (Fig. 2 and Supplementary Table S4 online).

### CR-GNB nosocomial infection incidence

Nosocomial CR-GNB infection was identified in 530 patients: 422 in high-risk departments and 108 in non–high-risk departments. The nosocomial infection incidence showed an overall downward trend (all *P* <0.001) in both the high-risk and non–high-risk departments. Compared with the previous year, the CR-GNB nosocomial infection incidence in high-risk departments decreased in 2018 and 2020 and in non–high-risk departments in 2019 (all *P* < 0.05) (Fig. 2 and Supplementary Table S5 online).

### Microbiological data

The detection rates (from both the high-risk and non–high-risk departments) of carbapenem-resistant *Klebsiella pneumoniae*, carbapenem-resistant *Acinetobacter baumannii* and carbapenem-resistant *Pseudomonas aeruginosa* decreased (*P* <0.001) (Fig. 2 and Supplementary Table S6 online). The resistance characteristics are shown in Supplementary Table S7 (online).

## Discussion

Chinese guidelines on interrupting CR-GNB transmission^7^ recommend various kinds of placement and cohorting despite there being few investigations assessing the effect of these interventions. In this study, the rate of use of type A and B placement in non–high-risk departments in 2018 was only 8.4%, whereas it exceeded 20% in high-risk departments and the CR-GNB nosocomial infection rate decreased only in high-risk departments. Compared with the previous year, the nosocomial CR-GNB infection incidence declined significantly in 2020 and 2019 in high-risk and non–high-risk departments in association with increased use of placement types A and B. Therefore, compared to type C or D, we conclude that patient placement types A and B may more effectively interrupt CR-GNB transmission. When a single room is not available, placement type B should be pursued.

This study had several limitations. The analysis included only a few variables hypothesized to influence nosocomial CR-GNB infection and colonization. In particular, the coronavirus disease 2019 (COVID-19) pandemic may have played roles in our findings in 2020 and 2021. Fewer patients were admitted to the medical wards, more protective equipment was used, and exhaustive environmental disinfection could be performed, which may have led to changes in hospital population and risk factors for CR-GNB. Finally, the active screening was mainly concentrated within the first 48 hours of admission, and the low rate of compliance with subsequent weekly screening limited our ability to assess acquisition of CR-GNB during hospitalization and thus to assess the impact of our interventions.

In conclusion, the intervention measures appeared to correspond with decreases in CR-GNB colonization acquisition and infection rates, and these interventions may be useful to decrease CR-GNB colonization and infection in resource-limited settings.

## Supplementary material

For supplementary material accompanying this paper visit https://doi.org/10.1017/ice.2023.20. click here to view supplementary material
