# Supplementary material for: Active screening and patient-placement and cohort-placement strategies to decrease carbapenem-resistant gram-negative bacilli colonization and infection in pediatric patients: A 5-year retrospective observational study in China
Source: Infect Control Hosp Epidemiol. 2023 Apr 24;44(10):1666–9. doi: 10.1017/ice.2023.20 (PMC10587376; doi:10.1017/ice.2023.20)
Supplement: Supplementary file 1 [file S0899823X2300020Xsup001.docx]

**Table S1. Percentage of patients with Carbapenem-resistant Gram-negative bacilli cared for using each of four isolation and cohorting strategies: 2018-2021(%)**

Percentages (Carbapenem-resistant Gram-negative bacilli positive patient number)

| Year | high risk departments^&^ | | | |  | Non-high risk departments^&^ | | | |
| --- | --- | --- | --- | --- | --- | --- | --- | --- | --- |
|  | N=3219 | type A/B* | type C* | type D* |  | N=729 | type A/B* | type C* | type D* |
| 2018 | 906 | 22.1(200) | 34.2(310) | 43.7(396) |  | 120 | 8.4（10） | 10.8（13） | 80.8（97） |
| 2019 | 816 | 24.0(196) | **39.2(320)** | **36.8(300)** |  | 227 | **15.9（36）** | **19.8（45）** | **64.3（146）** |
| 2020 | 578 | **35.1(203)** | **45.8(265)** | **19.1(110)** |  | 201 | 22.4（45） | **37.3（75）** | **40.3（81）** |
| 2021 | 919 | 34.5(317) | **22.3(205)** | **43.2(397)** |  | 181 | 26.0（47） | 42.0（76） | 32.0（58） |
| P^#^ |  | ＜0.001 | ＜0.001 | ＜0.001 |  |  | 0.001 | ＜0.001 | ＜0.001 |

*the 'single room placement ' (type A); the 'same room placement'(type B); the 'same area placement' (type C); no cohort placement (type D).

^#^ Comparison of patient placement type between years.

The bold:P <0.05 compared with the previous year

& High risk departments, including the pediatric intensive care unit, neonatal intensive care unit, neonatal and hematology departments; Non-high risk departments, other departments except for the high risk departments

**Table S2 Hand hygiene compliance rate, qualified rate of cleaning and disinfection effect (%)**

|  | 2017 | 2018 | 2019 | 2020 | 2021 | P |
| --- | --- | --- | --- | --- | --- | --- |
| Hand hygiene compliance rate (%) | 81.9（2198//2683） | 83.1(2938/3534) | 83.2(3155/3793) | 83.2(3271/3933) | 84.8(2779/3277) | 0.058 |
| Qualified rate of cleaning and disinfection effect (%) | 99.5(6353/6388) | 99.4(3659/3680) | 99.3(4315/4344) | 99.6(4708/4728) | 99.5(3598/3615) | 0.569 |

**Table S3 Compliance rate of CR-GNB active screening in high risk departments (%)**

| Rate (the number of patients screened / the number of patients eligible screened) | | | | | | | | | | | | | |
| --- | --- | --- | --- | --- | --- | --- | --- | --- | --- | --- | --- | --- | --- |
| Course of hospitalization | Upper respiratory tract | | | | |  |  | Intestinal tract | | | | | |
|  | 2017 | 2018 | 2019 | 2020 | 2021 | P^#^ |  | 2017 | 2018 | 2019 | 2020 | 2021 | P^*^ |
| ≤48h | 30.4 | 48.5 | 48.2 | 45.3 | 64.8 | <0.001 |  | 11.1 | 40.7 | 43.4 | 40.2 | 58.8 | <0.001 |
|  | (3594/11822) | (5391/11115) | (4396/9120) | （3340/7373） | （5219/8054） |  |  | (1317/11822) | (4523/11115) | (3960/9120) | （2962/7373） | （4731/8054） |  |
| 3-7d | 4.4 | 7.3 | 6.8 | 7 | 9.5 | <0.001 |  | 5.3 | 8.0 | 7.1 | 14.2 | 14.5 | <0.001 |
|  | (345/7806) | (668/9201) | (408/6000) | （392/5600） | （568/5979） |  |  | (414/7806) | (736/9201) | (426/6000) | （795/5600） | （867/5979） |  |
| 8-14d | 8.8 | 5.7 | 5.1 | 8.0 | 11.5 | <0.001 |  | 9.8 | 6.0 | 5.4 | 10.7 | 13.0 | <0.001 |
|  | (565/6422) | (536/9401) | (188/3720) | （274/3446） | （421/3676） |  |  | (617/6310) | (502/8430) | (206/3825) | （370/3453） | （472/3638） |  |
| ＞14d | 13.2 | 14.7 | 13.3 | 13.7 | 15.8 | <0.001 |  | 14.5 | 11.3 | 12.7 | 16.7 | 18.6 | <0.001 |
|  | (834/6303) | (1356/9200) | (479/3601) | （468/3400） | （573/3620） |  |  | (907/6223) | (950/8395) | (482/3782) | （571/3413） | （673/3607） |  |
| P^*^ | ＜0.001 | ＜0.001 | ＜0.001 | ＜0.001 | ＜0.001 |  |  | ＜0.001 | ＜0.001 | ＜0.001 | ＜0.001 | ＜0.001 |  |

^#^ Comparison of CR-GNB compliance rate between years

^*^ Comparison of CR-GNB compliance rate between hospitalization times

CR-GNB,Carbapenem-resistant Gram-negative bacilli

**Table S4 CR-GNB colonization prevalence in high risk departments (%)**

| Incidences (the number of patients with CR-GNB positive screened / the number of patients screened) | | | | | | | | | | | | | |
| --- | --- | --- | --- | --- | --- | --- | --- | --- | --- | --- | --- | --- | --- |
| Course of hospitalization | Upper respiratory tract | | | | |  |  | Intestinal tract | | | | | |
|  | 2017 | 2018 | 2019 | 2020 | 2021 | P* |  | 2017 | 2018 | 2019 | 2020 | 2021 | P* |
| ≤48h | 3.1 | 3.1 | 3.2 | 1.9 | 2.1 | <0.001 |  | 3.2 | 3.5 | 3.4 | 1.9 | 1.8 | <0.001 |
|  | (112/3594) | (165/5391) | (141/4396) | (62/3340) | (110/5219) |  |  | (42/1317) | (159/452) | (135/3960) | （55/2962） | (85/4731) |  |
| 3-7d | 5.8 | 4.8 | 4.4 | 3.3 | 3.9 | 0.508 |  | 8.5 | 7.9 | 6.6 | 5.2 | 6.3 | 0.142 |
|  | (20/345) | (32/668) | (18/408) | (13/392) | (22/568) |  |  | (35/414) | (58/736) | (28/426) | (41/795) | (55/867) |  |
| 8-14d | 16.8 | 6.9 | 5.3 | 5.1 | 3.3 | <0.001 |  | 13.3 | 7.2 | 7.8 | 8.1 | 8.5 | <0.001 |
|  | (95/565) | (37/536) | (10/188) | (14/274) | (14/421) |  |  | (82/617) | (36/502) | (16/206) | (30/370) | (40/472) |  |
| ＞14d | 20.4 | 13.9 | 9.4 | 9.6 | 9.2 | <0.001 |  | 17.9 | 15.6 | 12.1 | 11.9 | 8.2 | <0.001 |
|  | (170/834) | (188/1356) | (45/479) | (45/468) | (52/573) |  |  | (164/907) | (135/950) | (58/482) | (68/571) | (55/673) |  |
| P# | ＜0.001 | ＜0.001 | ＜0.001 | ＜0.001 | ＜0.001 |  |  | ＜0.001 | ＜0.001 | ＜0.001 | ＜0.001 | ＜0.001 |  |

^*^ Comparison of CR-GNB colonization prevalence between years

^#^ Comparison of CR-GNB colonization prevalence between hospitalization times

CR-GNB,Carbapenem-resistant Gram-negative bacilli

**Table S5** **CR-GNB nosocomial infection incidence (%)**

Incidences (number of new CR-GNB nosocomial infection cases /number of patients during the observation period)

| Year | high risk departments |  | Non-high risk departments |
| --- | --- | --- | --- |
| 2017 | 1.16(153/13190) |  | 0.12(51/40903) |
| 2018 | **0.89(111/12488)** |  | 0.09(39/43113) |
| 2019 | 0.87(104/11951) |  | **0.02(9/45147**) |
| 2020 | **0.28(26/9146)** |  | 0.01(5/34135) |
| 2021 | 0.29(28/9774) |  | 0.01(4/39856) |
| P^#^ | ＜0.001 |  | ＜0.001 |

^#^Comparison of CR-GNB infection incidence between years

The bold:P <0.05 compared with the previous year

CR-GNB, Carbapenem-resistant Gram-negative bacilli

**Table S6 Carbapenem-resistant Gram-negative bacilli detection rate (%)**

Rate (CR-GNB positive clinical isolates/ GNB-positive clinical isolates)

|  | 2017 | 2018 | 2019 | 2020 | 2021 | P |
| --- | --- | --- | --- | --- | --- | --- |
| CRKP | 33.4(215/644) | 23.6(127/538) | 13.8(81/585) | 8.9(36/403) | 11.0(51/464) | <0.001 |
| CRAB | 50.0(124/248) | 53.8(98/182) | 68.7(170/247) | 57.9(70/121) | 31.5(45/143) | <0.001 |
| CRPA | 30.6(83/271) | 40.3(123/305） | 25.8(73/283） | 19.7(47/238） | 10.7(25/233) | <0.001 |

CR-GNB,Carbapenem-resistant Gram-negative bacilli; GNB, Gram-negative bacilli;CRKP,Carbapenem resistant Klebsiella pneumonia;CRAB,Carbapenem-resistant Acinetobacter baumannii; CRPA,Carbapenem-resistant Pseudomonas aeruginosa

Table S7 Drug resistance rate of Carbapenem-resistant Gram-negative bacilli (%)

| Antibiotic name | CRE^&^ | | |  | CRPA | | |  | CRAB | | |
| --- | --- | --- | --- | --- | --- | --- | --- | --- | --- | --- | --- |
|  | ^#^high risk departments（n=611) |  | ^#^non-high risk departmentss (n=468) |  | ^#^high risk departments  (n=231) |  | ^#^non-high risk departmentss (n=123) |  | ^#^high risk departments (n=414) |  | ^#^non-high risk departmentss (n=108) |
| Cefoperazone/Sulbactam | 87.2 |  | 80.2 |  | 51.2 |  | 47.1 |  | 60.7 |  | 50.9 |
| Ampicillin/Sulbactam | 100 |  | 100 |  | - |  | - |  | 93.2 |  | 91.7 |
| Piperacillin/Tazobactam | 72.2 |  | 70.3 |  | 48.9 |  | 42.6 |  | 98.9 |  | 90.9 |
| Ceftazidime | 95.3 |  | 93.1 |  | 50.9 |  | 47.2 |  | 98.1 |  | 98.1 |
| Cefepime | 73.8 |  | 76.5 |  | 56.3 |  | 62.6 |  | 94.7 |  | 96.3 |
| Aztreonam | 86.3 |  | 78.9 |  | 61.3 |  | 58.1 |  | - |  | - |
| Ertapenem | 100 |  | 100 |  | - |  | - |  | - |  | - |
| Imipenem | 81.3 |  | 77.7 |  | 98.7 |  | 95.1 |  | 99 |  | 98.1 |
| Meropenem | 85.9 |  | 77.5 |  | 91.2 |  | 87.6 |  | 99 |  | 99.1 |
| Amikacin | 10.8 |  | 13.9 |  | 43.7 |  | 31.7 |  | 89.7 |  | 84.8 |
| Gentamicin | 30.4 |  | 37.8 |  | 22.5 |  | 23.6 |  | 86.9 |  | 86.1 |
| Ciprofloxacin | 22.9 |  | 60.8 |  | 44.7 |  | 37.8 |  | 96.5 |  | 85.3 |
| Levofloxacin | 29.3 |  | 45.8 |  | 29 |  | 30.6 |  | 37 |  | 27.8 |
| Trimethoprim/Sulfamethoxazole | 29.5 |  | 51.3 |  | - |  | - |  | 70.3 |  | 81.5 |
| ^#^Polymixin B | 0 |  | 0 |  | 3.3 |  | 10 |  | 0 |  | 10 |
| ^#^Tigecycline | 0 |  | 0 |  | - |  | - |  | 0.6 |  | 0 |
| Piperacillin | - |  | - |  | 52 |  | 49.2 |  | 98.2 |  | 94.4 |
| *Nitrofurantoin | 24.9 |  | 28 |  | - |  | - |  | - |  | - |
| ^#^Ceftazidime/Avibactam | 73.3 |  | 70 |  | - |  | - |  | - |  | - |

^&^The resistance rate of CRE isolates was 100% to Cefazolin,Cefuroxime,Ceftriaxone,Cefotaxime and Amoxicillin/Clavulanic acid in both high risk departments and non-high risk departments. The resistance rate to Cefoxitin,Cefmetazole and Cefotetan was 97.4% and 88.1%, 84.5% and 80.2% , 63.1 and 76.6% in high risk departments and non-high risk departments, respectively.

* Results of urinary tract isolates only; ^#^Incomplete data, only a few strains have been tested for drug sensitivity; -, indicates that the test was not conducted on this strain.

^#^ High risk departments, including the pediatric intensive care unit, neonatal intensive care unit, neonatal and hematology departments;Non-high risk departments, other departments except for the high risk departments

CRE,carbapenem-resistant Enterobacteriaceae; CRAB,carbapenem-resistant Acinetobacter baumannii; CRPA,carbapenem-resistant Pseudomonas aeruginosa
